# Supplementary material for: ‘once they see blood then the mood for sex is spoiled’ A qualitative exploration of female sex worker’s male client views of menstruation, sex during menses and the menstrual disc
Source: PLoS One. 2024 Dec 26;19(12):e0315383. doi: 10.1371/journal.pone.0315383 (PMC11670952; doi:10.1371/journal.pone.0315383)
Supplement: S1 File — (PDF) [file pone.0315383.s001.pdf]

Community Men *To be held after FGDs with women, so that taboos and perceptions may be checked

- **Overview/ Warm up / knowledge of menses -**What do you know about menses? Probe (after they have said) purpose, ages affected, frequency, Where did you get your information? What do you think about the information you received? Do you think men in general know about (know enough about) menstruation? Do you think men should know about menstruation? Why / not? What do they need to know? How should they get this information?
- **Taboos -** What do you think about women when they are menstruating? Are there things they should do / should not do? What about womens hygiene when they are menstruating? What should they do? Anything they should not do? Is there anything that might help to make men feel differently about menstruation in general? What?
- **Men v women responsibility** If a man is married or has a partner, who should be responsible for making sure they have the right products to wear during their menses? Should a husband or partner know when their wife is menstruating? Why / why not? Do you think a husband or partner should talk to their wives or partners about menstruation? Why / not?
- **Sex during menses -**What do men think about having sex during menses? Is there any problem with this? What? Why? Do men have a different type of sex if the woman is menstruating? What / why? Do men's hygiene practices differ if a woman is menstruating? In what way? Why? Is there anything that might help to make men feel differently about having sex during menstruation?
- **Thoughts on menstrual blood-** What are your thoughts about a woman using tissue or something similar inside herself to stop the blood flow during sex? Is menstrual blood the same as normal blood? Why / not? How would men react to getting menstrual blood on themselves?
- **Sex during menses – differences between type of partner -** Is there any difference between having sex with a wife / partner or a sex worker during menses? Why / not? Do you think a wife / partner should tell the man if they are going to have sex, that she is menstruating? Why? What kind of reaction would this get? What if this were a sex worker – would it be different? Why? What if a wife / partner didn't tell a man she was menstruating and they had sex, then he realised? How would he react? What if this were a sex worker, how would he react? If men react badly, is there anything that might help them to react differently?
- **Menstrual cup information** Have you heard about the menstrual cup? What are your thoughts about it? Is there anything else you need or want to know about it?
- Menstrual hygiene explanations and demonstrations (10-15 minutes). To overview brief explanation, with product demonstration on menstrual management products: Pads (reusable, disposable), Inserted absorbents, menstrual cups.
- **Continue Discussion:** How do you think information is best given to men about menstruation and how women manage it? What about the menstrual cup? (who from, how, when….)

- **Sex with a menstrual cup?** What are your thoughts about a man having sex with a wife / partner whilst she is wearing it? (more likely to / avoid / why?) Would men want to know if she was wearing it? Why / not? What are your thoughts about a sex worker wearing a cup during sex – is this different? if it were a sex worker – would that be different? Why? Would men want to know if a sex worker was wearing a cup? Why / not?
- **Use of menstrual cup in prevention of HIV and pregnancy -**added as it kept coming up
- **Round up -** What do you think about this discussion we have just had? Is there anything that you think we should have talked about but did not?
