# Supplementary material for: ‘once they see blood then the mood for sex is spoiled’ A qualitative exploration of female sex worker’s male client views of menstruation, sex during menses and the menstrual disc
Source: PLoS One. 2024 Dec 26;19(12):e0315383. doi: 10.1371/journal.pone.0315383 (PMC11670952; doi:10.1371/journal.pone.0315383)
Supplement: S2 File — (PDF) [file pone.0315383.s002.pdf]

Power Health - Men's Focus Group: Brief Education on Menses and Menstrual Hygiene Management

Introduction

As part of the male clients focus group discussion, after getting initial feedback on what men know about menses, how women manage them, and their thoughts about sex during menses, there will be a 10-15 minute interlude in which participants are provided with information regarding: (1) the menstrual cycle, (2) menstrual hygiene management. The purpose of this to provide standardized information and introduce men to menstrual cups, to facilitate the remainder of the discussion.

Overview of Educational Session

1. Brief summary of the menstrual cycle (3 minutes)
2. Explanation of menstrual hygiene management
    a. Privacy and hygiene needs, Disposal of materials, Stigma (5 minutes)
    b. Materials for management (7 minutes)
        i. This will include a demonstration with pads, tampons, and lastly menstrual cups

Men will be given a one-page handout to read and take with them if they wish

Materials used in session:

- Cotton wool, rag, tissue
- Pads (reusable and disposable)
- Tampons
- Menstrual cup
- Laminated menstrual cup card
- 3D uterus model

Script

1. Menstrual cycle basics

Instruction (not read to participants): *The initial question in the FGD asks men what they know about menses: they may not know much, or they may know a lot, there may be gaps to fill or misconceptions to dispel. Begin the education by recapping what was said **accurately** during this initial discuss, and build from there as needed, using the information from the script. It is worth bearing in mind the range of participants and considering those who have the least knowledge. So even if some men appear to know a lot, there may be some who appear to agree, but don't really know so much.*

In the beginning, when I asked you what you know about the menstrual cycle, you told me some correct things, such as [***paraphrase from discussion***]. I will add some more information here, so we are on the same page in understanding women's menses.

[Use 3D model to explain female anatomy of ovaries, uterus, vagina]
"Around every 23 to 35 days, the uterus develops a lining intended to nourish and protect a fertilized egg (fetus). The fertilized egg must attach itself to the lining in order to grow." When the egg is not fertilized, the lining is shed along with the unfertilized egg and tissue with blood and vaginal secretions. This regular shedding of the uterine lining is called menstruation, or a period. This is a normal function of being a woman, and menstrual blood is not dirty or unhygienic. A woman's period usually lasts 3-7 days, but for some it could be shorter or longer. During the period, how much blood does the woman lose? Usually 2-3 tablespoons, though it could be as little as 1 tablespoon or as much as 5 tablespoons and this is normal. Many women, but not all, have symptoms before and during their menses. This could include abdominal cramps, headaches, sore breasts, bloating, diarrhea, fatigue and even food cravings, mood swings, and acne. Cramps are caused by the contractions that cause the uterine lining to shed. Food cravings, mood changes, and acne are caused by the changing hormone levels (estrogen and progesterone).

2. Menstrual hygiene management

Women have many ways to manage their menses. Some of these are safe and some are unsafe. Let's fist talk about what women need for good menstrual hygiene management – it is more than just pads and tampons.

First, women need a private space to apply and change their menstrual materials. Sindio? Does anyone disagree? That private space – whether it is a choo or other space, should be clean and have a light for evening hours and should have a door. Ideally, the door is locking.

Secondly, women must have a way to practice good hygiene. They should have soap and water to wash their hands before and after touching their vaginas or removing and inserting the menstrual products. This is necessary to prevent infection.

Thirdly, women must have somewhere to dispose of their used materials. For example, imagine, a woman has used a pad, and there is nowhere to throw it. What should she do? Should she fold it up, with the blood, and carry it with her? Do you think that would be a good idea?

Lastly, women need appropriate products to manage their menses, and I will show and explain to you about these. Just as I am going to give you the information, girls and women must be given the same education and information. But many parents, schools, and health care providers don't teach this to girls, and there are taboos on talking about menses. Girls and women without knowledge may sometimes use unsafe materials to manage menses. Or maybe they don't have the money to buy these things. Sometimes parents and partners buy them, but sometimes women must resort to makeshift materials.

Here are some makeshift materials: **[show cotton wool, tissues, and rags/cloth]**
In addition to these, some women may resort to sponges or mattress stuffing. These are not good for managing menses, sometimes they do not absorb the blood well and can leak. This can cause a woman to worry that others will notice leakage and she may feel ashamed. These unhygienic materials can harm the health of the woman. Inserting these objects to the vagina can lead to infections and injuries.

Now we will talk about some safer ways to manage menses.

These are pads – some are reusable and some are disposable**.** **[Show pads]**
The disposable pad has an absorbent side and a sticky side. The sticky side is placed in the underwear to keep it in place as the woman moves around. As blood flows from the vagina, it is absorbed in the material. Each pad can be worn up to 4-6 hours. The disposable pads can be purchased in supermarket or other stores. The cost of these is approximately 100-250 shillings. As we talked about, women menstruate on average every 4 weeks, though 3-5 weeks is normal. So they are spending this money regularly, which could be 2000-4000 KSH in a year. And they will menstruate for 30 years or more. What else could a woman buy with that money?

Sometimes to save money, or if they are unable to change, perhaps if there is nowhere safe or private for them, women and girls may wear a pad too long. This can lead to leaking of blood, odors, or chaffing around their vagina. Wearing the pad too long can even increase the risk of vaginal infections.

Because disposable pads are costing, some women use reusable pads. **[Show reusable pad]**
The reusable pads usually have a snap or button to fix to the underwear. Now the reusable pads save money, but they have challenge of having to be cleaned. They may be difficult to clean properly and dry thoroughly due to lack of privacy. The reuse of unclean pads can lead to increased risk of vaginal infections. Also, many women and girls complain that the reusable pads smell.

Next, we have tampons. **[Show tampon]**
Tampons are an absorbent material that is inserted into the vagina. Usually, the woman pushes it in with her finger or sometimes there is an applicator. If it is inserted properly, it cannot be felt so is very comfortable. Tampons can be purchased at the super market for 500-1000 shillings, or approximately 6000-15000 shillings per year for women who need to use more than one box per period. Tampons can be worn up to 6 hours. Wearing them longer can lead to increased risk of infection. If a tampon is left very long, it can lead to a life threatening infection called toxic shock syndrome. Tampons, like disposable pads, also need to be disposed of properly.

Finally, we have menstrual cups. **[Show menstrual cup]**
Menstrual cups are made of medical grade silicone. They are inserted into the vagina to collect the blood. **[Show laminated card as pass the cards around]**

They can be worn up to 12 hours and are NOT associated with increased risk of infections for women. One menstrual cup can last up to 10 years if it is taken care of.

Menstrual cups are widely available in the markets in the US, Europe, Canada, India and other countries of Asia and their safety is approved by numerous regulatory bodies. In Kenya, there are a few brands available – like Ruby cup - but they are not yet well known and are mostly available in big cities or specialty shops. One reason menstrual cups are not yet widely available in Kenya is the cost. Menstrual cups are very expensive. They can cost upwards of 1,500 shillings for one. What can 1500 shillings buy?

But, a menstrual cup can last 10 years. Over time, this saves a girl and woman money because she doesn't have to keep buying pads or tampons. And, as you can see, this menstrual cup is small. If a woman is going somewhere, she doesn't have to carry with her several bulky pads or tampons. She also doesn't need to find a place to dispose of it when done. She can just empty the blood in the loo, and reinsert it. At the end of her period, she may sterilize the cup by steeping in boiling water.

A menstrual cup does not protect against STIs, HIV or pregnancy and therefore should not be used for the same. It is used just as a sanitary towel and the other items that women use during menses.

Some men do not like to have sex during a woman's menses. Even though menstrual blood is a normal bodily fluid, they fear the blood – that it is dirty or could cause infection [***or other paraphrase from prior discussion***], or they prefer the vagina to be drier. To manage the menses during sex, some women insert cotton wool or sponge or tissue into their vaginas during sex to absorb the blood and keep the vagina dry. This can increase the risk of injuries to their vagina or infection.

Now, some menstrual cups, like the one I am showing you, can be worn during sex. These types of menstrual cups are safe for both the man and the woman. Most often, the man cannot feel the menstrual cup with his penis. But sometimes, they may feel it. There have been no reports of pain or injury to the man from menstrual cups being worn during sex.

Now, what are your questions about the menstrual cup?

Any questions about menstrual hygiene management?

To recap before we move on, most women and girls menstruate and this takes place approximately every 4 weeks, though some women may have a shorter or longer cycle. Bleeding lasts usually 2-7 days, though for some women it may be shorter or longer. Many women experience cramping, bloating, headaches, or other changes with changing hormone levels and as the uterus contracts to shed the lining during menses. On average, menses are a few tablespoons of blood, but the blood must be managed with hygienic products. To manage their menses well, women and girls need privacy, safety, soap and water for hygiene, information and education, and good quality menstrual products. Poor quality products increase their risk for vaginal injuries and infections.

Now, we will continue with our Discussion.
