# Supplementary material for: ‘once they see blood then the mood for sex is spoiled’ A qualitative exploration of female sex worker’s male client views of menstruation, sex during menses and the menstrual disc
Source: PLoS One. 2024 Dec 26;19(12):e0315383. doi: 10.1371/journal.pone.0315383 (PMC11670952; doi:10.1371/journal.pone.0315383)
Supplement: Attachment — Submitted filename: Response to reviewers.docx [file pone.0315383.s004.docx]

*‘once they see blood then the mood for sex is spoiled’* A Qualitative Exploration of Female Sex Worker’s Male Client Views of Menstruation, Sex during Menses and The Menstrual Disc

Dear Editor and Reviewer 3,

Thank you very much for taking the time and effort to review our paper along with the previous reviewer feedback. We have outlined below the changes we have made in accordance with both of your suggestions. We hope that our amendments meet with your approval.

**Line : 122 - page 6.

“this method allows group dynamics…” - Maybe just a rephrase is needed here to emphasise that the group dynamics enabled (the bonding experience)**

Thank you for your suggestion. We have amended as per below:

‘This method can facilitate a productive discussion through group dynamics, and lead to a bonding experience for participants who we hypothesised, may have felt embarrassed or reticent in talking about menstruation in a one-to-one situation with a researcher.’

**page 26 - conclusion:
Would it be possible to add an extra sentence or even just a few words linking back to the focus on FSW’s male client views of menstruation? The introduction, results, and discussion clearly articulate the intersecting emphasis/gap/significance. It would be great to see it mirrored more here.**

We hope the following amendments have been able to link our conclusions back to the focus as suggested. The conclusion now reads:

‘Our study has added to the limited research on men’s knowledge and attitudes toward menstruation focusing on their views of sex during menses with FSW to help understand how it may contribute to unsafe or risky menstrual hygiene practices that these women undertake. This research highlights men’s lack of knowledge and largely negative perceptions about the topic, including towards sex during menses. Researchers, policy makers, politicians alike have been calling for increased awareness of boys and men around menstruation. We echo this need, calling for accurate information including on school curricula, and through faith teaching where appropriate, to address the current lack of awareness that contributes to stigma and shame, and perpetuation of menstrual taboos. This research illuminates men’s need for education around reproductive health in general, noting that participants were receptive to, and curious about a reusable menstrual disc that can be worn during sex, which suggests that this has potential to be adopted by FSW with minimal adverse client reaction. Further it highlights possible partner support for women considering adopting a menstrual disc in the future. These study findings can be used to inform successful menstrual health programme implementation for FSW in the future’.
